# Impact evaluation of contracting primary health care services in urban Bangladesh

**DOI:** 10.1186/s12913-019-4406-5

**Published:** 2019-11-21

**Authors:** Manuel Leonard F. Albis, Subrata K. Bhadra, Brian Chin

**Affiliations:** 1 School of Statistics, University of the Philippines, Quezon City, NCR Philippines; 2 National Institute of Population Research and Training, Dhaka, Bangladesh; 3 Asian Development Bank, Manila, Philippines

**Keywords:** Contracting, Primary health care, Maternal and child health, Urban health, Bangladesh, Impact evaluation, Propensity score matching, UPHCP-II

## Abstract

**Background:**

The Urban Primary Health Care Project (UPHCP) was implemented by the Government of Bangladesh in response to rapid urbanization and growing inequalities in access to and quality of primary health care. The goal of the project was to improve health status of the urban poor living in city corporations and municipalities through the provision of health care services by NGOs that are contracted through public-private partnership. The first phase of the project started in 1998 and the project is currently in its fourth phase covering more urban areas than the first three phases. This study evaluates the impact of the second phase project (UPHCP-II) on health outcomes, mainly child diarrhea, acute respiratory infection, antenatal and postnatal care, skilled birth attendance, breastfeeding prevalence, contraceptive prevalence, sexually transmitted infections, and HIV/AIDS awareness.

**Methods:**

The effect of the project was estimated through propensity score matching between project and non-project areas comparing baseline and endline surveys over a six-year period from 2006 to 2012. An innovation of this study is the recalibration of the sampling weights that allows the use of these two independent surveys in impact evaluation.

**Results:**

Over the six-year period, UPHCP-II improved the health status of the population in project areas compared to non-project areas. The study found significant improvement in health outcomes in terms of reduced diarrhea and acute respiratory infection in children, which explains the downward trend in child mortality rate. Moreover, the project also improved antenatal care and skilled birth attendance. Contraceptive prevalence and HIV/AIDS awareness and avoidance increased, and sexually transmitted infections decreased.

**Conclusions:**

UPHCP-II was effective in achieving its health outcome targets, while previous studies show that it was efficient in the delivery of health care and clients were highly satisfied because health facilities were in close proximity, and doctors and staff were perceived as responsive in delivering high quality of care. The results of this study could help inform future design and implementation of urban health interventions that involve contracting primary health care service delivery in Bangladesh and other similar settings.

**Electronic supplementary material:**

The online version of this article (10.1186/s12913-019-4406-5) contains supplementary material, which is available to authorized users.

## Background

Bangladesh’s 26.7 million urban population in 2011 is projected to be 100 million by 2050 [1]. Because of rapid urbanization, the proportion of urban population is expected to exceed the rural population by 2040 [2]. The large influx of people in cities expanded the slum areas without basic infrastructure and social services [3]. Moreover, the search for employment to escape poverty, natural disasters, river erosion, and multiple effects of climate change drives rural to urban migration, resulting in the growing number of impoverished city dwellers. The health status in urban slums is often worse than in rural areas. For example, the mortality rate for children under 5 in urban slums was 91 per 1000 live births in 2006 [4], compared with 77 per 1000 live births in rural areas in 2007 [5].

While the urban poverty incidence decreased from 28.4% in 2005 to 21.3% in 2010, the non-negligible proportion of subsistence poverty at 7.7% in 2010 [6] means that a sizable poor population still have limited access to essential health care, compounded by their lack of health awareness. Because the poor have a small asset base, their provision for health welfare is sensitive to their daily income and vulnerable to economic shocks. Poor health limits productivity and constrains their income gains, preventing the impoverished from escaping poverty. Rapid urbanization causes challenges to urban health regarding poor sanitation and hygiene, which increases the burden of communicable diseases. Pollution in urban areas also compromises the health and welfare of city dwellers. Poor quality of housing also contributes to the hazards that poor urban households face. However, due to the heterogeneity of the urban population, poverty targeting is challenging. Even affluent households, based on their housing characteristics and ownership of durable goods, reside in poor neighborhoods. Public health facilities could not efficiently adjust to the fast-paced increase in urban population, sacrificing effective health care services. There is also the possibility that poor households live near affluent neighborhoods, thus establishing health facilities near poor neighborhoods might not be accessible to all poor households. Because of higher living standards in urban areas, the cost of curative care takes a large proportion of poor households’ income. The double burden of disease, the combined burden of communicable and non-communicable diseases, is a pressing problem for the urban poor in slum areas due to infectious disease and accidents in overcrowded living conditions, and mental ill-health from social fragmentation and weak community support mechanisms (relative to rural areas) [7].

The Urban Primary Health Care Project (UPHCP) was developed, financed, and implemented by the Government of Bangladesh (with financial and technical support from Asian Development Bank and other development partners). The project had a design of contracting primary health care services to non-government organizations (NGOs) to improve and support urban health across Bangladesh amidst the growing urban population and increasing difficulties in unaided access to health care of the urban poor. UPHCP started in 1998 and covered the city corporations of Dhaka, Chittagong, Khulna, and Rajshahi and demonstrated increased maternal and child health care utilization with improved equity among the poorest half of the population [8, 9]. The Second Urban Primary Health Care Project (UPHCP-II) (2005-2012), which is evaluated in this study, expanded coverage to include two additional city corporations, Barisal and Sylhet, and five municipalities, namely Bogra, Comilla, Sirajganj, Madhabdi, and Savar. The third phase project known as the Urban Primary Health Care Services Delivery Project (UPHCSDP) (2012-2018) covered 10 city corporations and four municipalities (more urban areas compared to the first two phases), and the ongoing fourth phase (UPHCSDP-II) (2018-2023) covers 12 city corporations and 13 municipalities (more urban areas than the first three phases).

UPHCP-II provided primary health care (PHC) through partnership agreements, expanded the network of urban PHC infrastructure, and provided capacity building to support project implementation and operationally relevant research. UPHCP-II aimed to improve equitable access and use of urban PHC services in the project area, with a focus on the poor; to improve the quality of PHC services in the project area; and to improve the cost-effectiveness, efficiency and the institutional and financial sustainability of PHC, with at least 30% of all PHC services provided for the poor.

The UPHCP-II modality of contracting primary health care services to NGOs follows the first phase’s design [9–15]. UPHCP-II financed 24 project areas, each covering 200,000 to 300,000 people, and established comprehensive reproductive health care centers (CRHCC) that provide emergency obstetric care, newborn care, and other specialized services. At least one PHC center catering to 30,000 to 50,000 people, and at least one satellite or mini-clinic per 10,000 people was established in each project. At project completion, 24 CRHCC, 161 PHC centers, 24 voluntary counseling and confidential testing centers for HIV/AIDS, and 24 primary eye care centers were established.

A set of health care services under UPHCP-II, called the essential services package plus, included immunization and growth monitoring of children; micronutrient support for malnutrition; family planning; prenatal, obstetric and postnatal care with special attention to prevent eclampsia; sexually transmitted infections, and HIV/AIDS; other reproductive health; and child health services. The project also included systematic case management of pneumonia and diarrhea in children; health education; sanitation, safe water and waste disposal; case management and services dealing with tuberculosis, leprosy, malaria, filarial and visceral leishmaniasis; and management of emergency cases.

UPHCP-II provided free health services and medicines to the poor, who were identified through household poverty surveys. Health entitlement cards (red cards) were given to the identified poor households and their members for free health services. Approximately 32% of each major type of service was provided to a poor client; non-poor or non-entitlement card holders pay subsidized rates [16].

UPHCP-II also included a behavior change communication and marketing (BCCM) component, which educated the urban population in order to increase their knowledge, improve their attitude, behavior, and practices related to health. BCCM activities included dissemination of information through posters, stickers, billboards, radio programs, and TV serials. The UPHCP-II rainbow logo clearly identified health facilities under the project, and thus established brand recall. Included in the communication program were pregnancy-related knowledge, awareness of communicable and non-communicable diseases, family planning methods, antenatal and child care, vaccination, violence against women, and others. Seminars and trainings were conducted to sustain effective and efficient delivery of health care, and capacity-building activities focused on pro-poor and gender-sensitive targeting and monitoring [17].

This study evaluates the impact of UPHCP-II on selected health outcomes using household surveys. The endline survey was collected under the comprehensive monitoring and evaluation component of UPHCP-II. The baseline survey used was not originally intended for evaluating UPHCP-II and has a different domain structure from the endline survey, as discussed in Section 2. Nonetheless, the two surveys have similar questionnaires, implying that outcome variables can be calculated using the same definition. The innovation introduced by this study is the recalibration of the sampling weights that allows the use of these two independent surveys in impact evaluation.

The effect of the project was estimated through difference-in-differences of health outcome indicators with propensity score matching between designated project and non-project areas. The results of the study can help inform implementation of ongoing urban health interventions and design of future ones in Bangladesh and other settings involving contracting primary health care. This study demonstrates the possibility of assessing the impacts of health projects with no baseline (or endline) surveys by utilizing standard demographic and health survey (DHS) type questionnaires as ex-ante measures of health status.

## Methods

UPHCP-II’s monitoring and evaluation system involved facility-based routine service delivery statistics, independent quality assurance assessments, facility-based surveys including client satisfaction, household poverty surveys, and baseline and endline surveys of urban households; all of which contributed to continuous improvement in the delivery of health services to intended beneficiaries throughout the project period. This impact evaluation uses the Urban Health Survey (UHS) 2006 (baseline) and the UPHCP-II Endline Survey 2012 (endline). Refer to [4, 18] for details regarding the baseline and endline surveys, respectively.

Both baseline and endline surveys have a DHS-type questionnaire that measures information on child and maternal health status, and other relevant health outcomes. The household surveys consist of a women’s questionnaire that captured the socioeconomic, demographic, and health-related information. Within the women’s questionnaire is a section of all the respondent’s children. The sample includes households in UPHCP-II project and non-project areas of Dhaka, Chittagong, Rajshahi, Khulna, Barisal, and Sylhet city corporations; as well as other municipalities. Households were identified as project areas (PA) following the same project areas identified in the second phase of the project, while non-project areas (NPA) are areas not covered by the project. The baseline and endline surveys used multi-stage cluster sampling that gathered information from 12,069 and 20,868 households, respectively. The similarity in the sampling design is an advantage when using two unrelated household surveys to measure the impact of the project. The sampling weights of the baseline were recalibrated to match the sampling design of the endline survey thereby making the two surveys comparable for impact evaluation.

### Recalibration of the baseline sampling weights

**Fig. 1:**
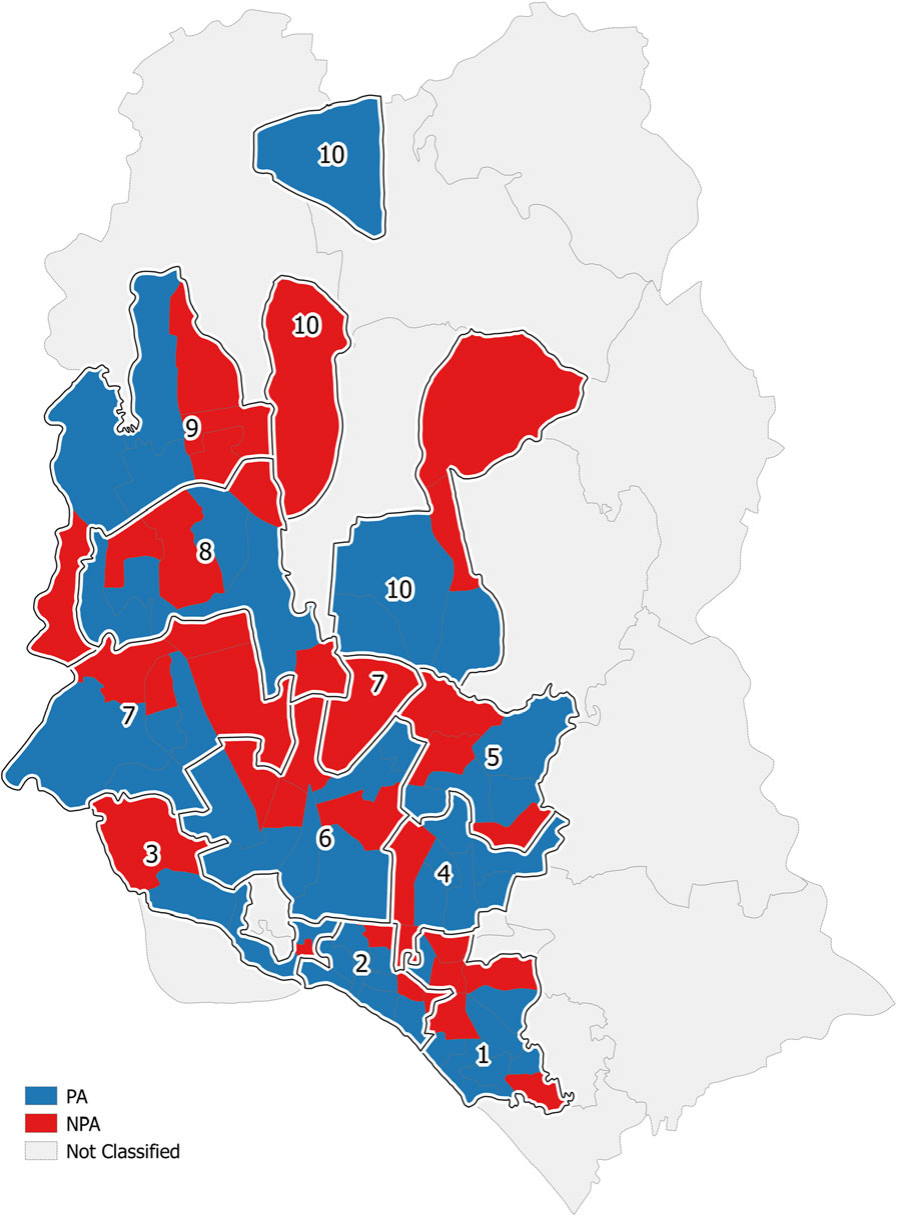
Sample Map of Dhaka City Corporation Showing Project and Non-Project Areas. Note: PA – project area; NPA – non-project area; 10 wards of Dhaka City Corporation in 2012 shown. Source: Authors’ own figure

UHS 2006 was used as the baseline survey due to the lack of a proper baseline survey for the project. Its data was transformed to follow the sampling design of the UPHCP-II endline survey. The survey domains are PAs and NPAs in Dhaka City Corporation (DCC), other city corporations (OCC), and municipalities. Figure 1 illustrates the PAs and NPAs in DCC as an example. There are 20 survey domains in DCC, 24 in OCC, and 16 in municipalities.

**Fig. 2:**
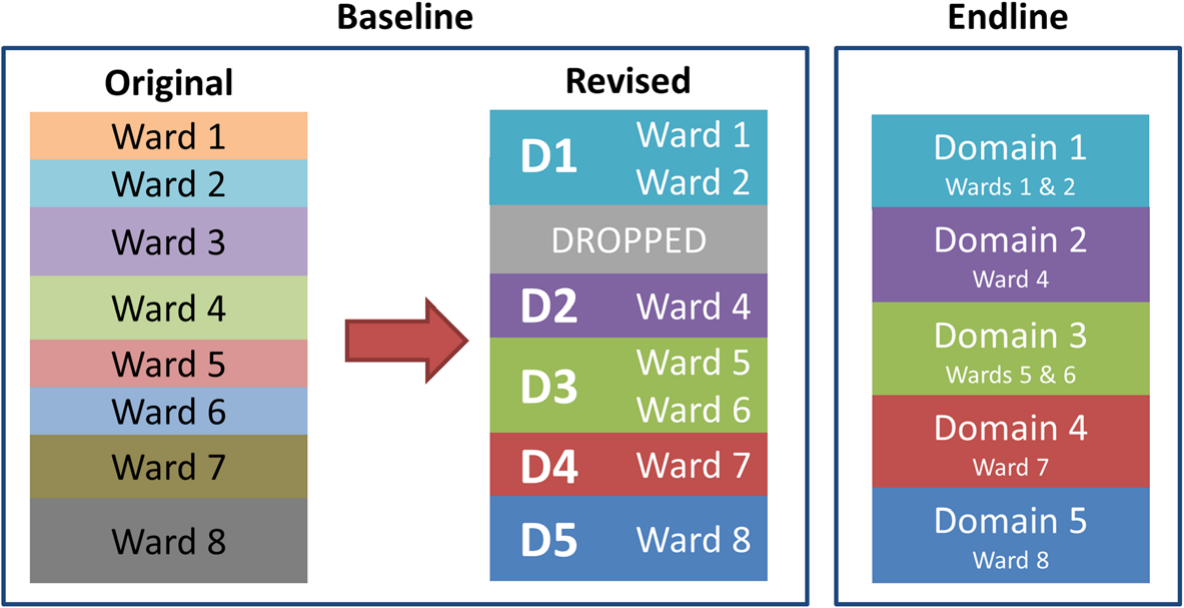
Recalibration of Baseline Survey Sampling Weights

The endline was used as a template for the baseline in updating and realigning the sampling weights as shown in Fig. 2. Suppose that Domain 1 of the baseline contains eight wards belonging to the first five domains of the endline survey. The baseline was transformed to mirror the domain structure of the endline survey; wards not included in the endline were dropped. Households in the baseline and endline domains were made comparable to be of the same geographic area and have consistent exposure to the project.

To further ensure comparability, the baseline survey weights were recalculated by assuming the sample selection procedure was that of the endline survey. The rationale is that the sampling weights reflect the sampling design of the survey, thus recalculating the sampling weights restructures the sampling design to make the two surveys comparable. Sample sizes in estimating the health outcome indicators are reported in Additional file 1: Appendix 1. The estimated health outcome indicators for the baseline and endline surveys in both PA and NPA are reported in Additional file 1: Appendix 2.

### Estimating project impact

The health outcome indicators examined include under-5 mortality rate (U5MR); child, infant and neonatal mortality; indicators of child nutrition such as stunting (height-for-age), wasting (weight-for-height) and underweight (weight-for-age); breastfeeding rates; prevalence of childhood diarrhea; and acute respiratory infection (ARI). Maternal health indicators examined include antenatal (ANC) and postnatal care (PNC); skilled birth attendance; contraceptive prevalence; sexually transmitted infection (STI); and HIV/AIDS awareness and avoidance.

Propensity score matching (PSM) was applied to health outcome indicators by matching using household and individual characteristics by survey period between PAs and NPAs. Correcting selection bias is the main objective of this approach. Treatment effects were then estimated between the baseline and endline surveys using matched difference-in-differences. The sample size per indicator varies due to the differences in characteristics of the target respondents, and only indicators with adequate sample size were evaluated.

The propensity scores of individuals in a project area were based on observed characteristics that are not affected by the treatment. It is assumed that only the observable characteristics could affect program participation (conditional independence assumption). A sizable overlap between the propensity score distributions of PA and NPA (common support assumption) is needed to ensure matching. Balancing tests were performed to check for the quality of matching. The propensity score conditional to observed characteristics was estimated by the logit model given by:

1 $$ P\left({T}_i=1|{\boldsymbol{X}}_i\right)=\frac{\exp \left({\mathbf{X}}_i\upbeta \right)}{1+\exp \left({\mathbf{X}}_i\upbeta \right)} $$

where *T*_*i*_ is the dummy for PA (treatment group) for the *i*^*th*^ individual, **X**_*i*_ is a vector of observed characteristics, and **β** is the vector of coefficients in the logit model.

The individuals between PAs and NPAs were matched using the nearest-neighbor matching algorithm satisfying the assumptions of conditional independence and common support. Average treatment effect on the treated (ATT) was calculated by matched difference-in-differences for two-period cross-sectional data [19]:

2 $$ ATT=\frac{1}{n_{T_2}}{\sum}_{i\in {T}_2}\left[{Y}_{i2}^T-{\sum}_{j\in {C}_2}\omega \left(i,j\right){Y}_{i2}^C\right]-\frac{1}{n_{T_1}}{\sum}_{i\in {T}_1}\left[{Y}_{i1}^T-{\sum}_{j\in {C}_1}\omega \left(i,j\right){Y}_{i1}^C\right] $$

where *ω*(*i*, *j*) is 1 if individual *j* in NPA is matched with individual *i* in PA, and zero otherwise under the nearest-neighbor matching approach. Estimated project effects are in terms of percentage point change.

Equation 2 performs matching separately for the baseline and endline surveys. A logit model for the propensity scores was first estimated for the endline. Variables satisfying the balancing test in the endline were also the ones used as matching variables in the baseline. The matching procedure was done separately for women and children. Applying eq. 2 to child- and women-related indicators, four matching procedures were performed: (1) children in the baseline, (2) women in the baseline, (3) children in the endline, and (4) women in the endline.

The variables that were used for matching the women are (1) wealth index, (2) age, (3) parity, (4) highest educational attainment, (5) religion, and (6) major geographic grouping (Dhaka city corporation, other city corporations, and municipalities). For matching the children, variables used were (1) age of child, (2) gender of the child, in addition to the women variables given above. The treatment group is a sample of individuals from designated PA and not restricted to individuals who have accessed UPHCP health facilities. The estimated logit models are reported in Additional file 1: Appendix 3, with summary statistics of the propensity scores in Additional file 1: Appendix 4. Results of the balancing test and graphs of the propensity score distributions are in Additional file 1: Appendix 5.

Health indicators such as U5MR and total fertility rate (TFR) are aggregated statistics, in which project impacts cannot be evaluated by the individual-specific nature of PSM. Moreover, impact evaluation was performed only on indicators with sufficient sample in both treatment and control groups, thus nutritional status was not included due to inadequate sample in the control group.

## Results

The baseline surveyed 14,191 women, while the endline surveyed 21,269 women respondents. In matching the sampling design between the two surveys, individuals located in the specified PA and NPA in the endline survey are the ones included in the analysis; 2405 individuals or 17% of the sample were outside the designated PAs and NPAs in the baseline. The total number of individuals in PAs after matching the sampling designs of the surveys is 7680 and 9438 for the baseline and endline, respectively (Table 1).

**Table 1:** Number of Individuals Per Area After Matching Sampling Designs

Area	Baseline		Endline	
	Project Area	Non-project Area	Project Area	Non-project Area
Dhaka	2046	1769	3314	3320
Khulna	798	794	711	707
Rajshahi	551	349	746	711
Barisal	398	215	347	341
Sylhet	299	234	363	387
Chittagong	3434	334	1092	1143
Comilla	61	28	375	373
Municipalities	93	182	2490	2622
Total	7680	3905	9438	9604

### Neonatal, infant, and U5MR

**Fig. 3:**
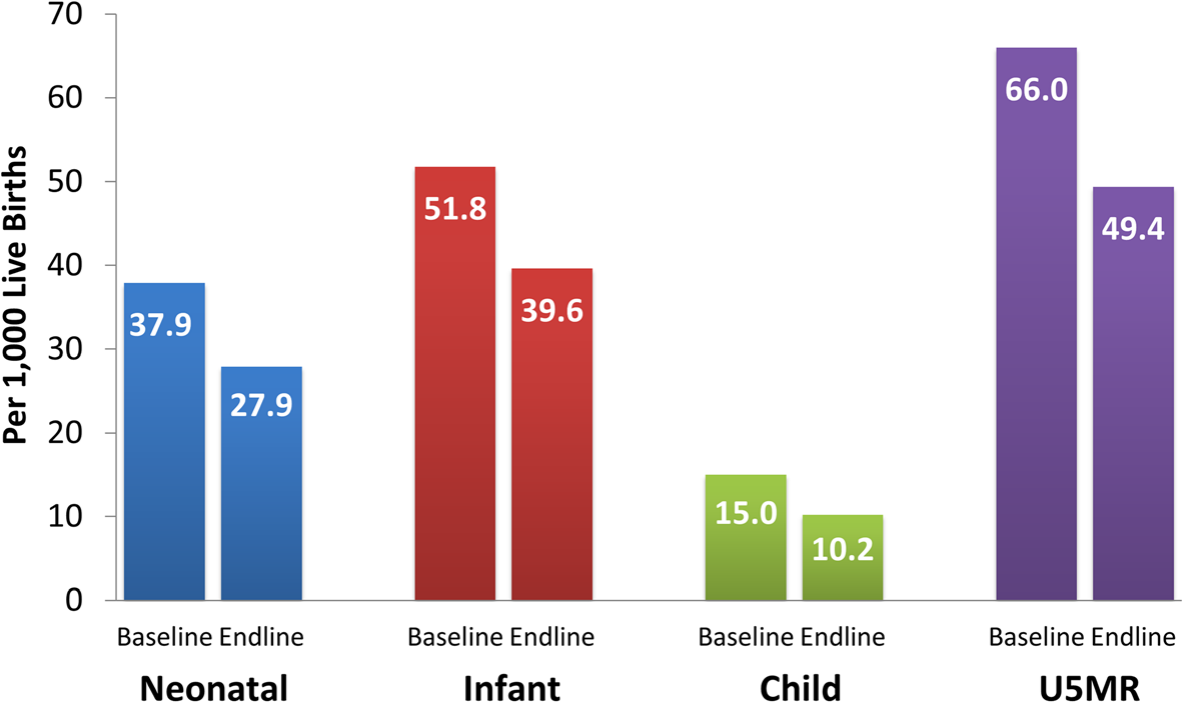
Trends of Child Mortality Rates in Project Areas

Analysis of neonatal, infant, and U5MR compares rates at baseline and endline. Neonatal mortality rate in PA decreased from 37.9 per 1000 live births in the baseline to 27.9 per 1000 live births in the endline (Fig. 3); a decrease of 26.4% or 10 per 1000 births. Infant mortality rate dropped from 51.8 to 39.6 per 1000 live births, resulting in a decrease of 23.6% or 12.2 per 1000 births. Child mortality rate decreased from 15.0 per 1000 live births in the baseline to 10.2 per 1000 live births in the endline; a 32% or 4.8 per 1000 births reduction. The under-5 mortality rate (U5MR) of 66.0 per 1000 live births in the baseline decreased to 49.4 per 1000 live births in the endline; a reduction of 25.2% or 16.6 per 1000 births.

In terms of equity, U5MR in the baseline are 98.5 and 20.8 per 1000 live births in the lowest and highest wealth quartile, respectively; while in the endline these are 46.9 and 45.5 per 1000 live births. Over the 6-year period, the differential in U5MR between the lowest and highest wealth quartiles reduced from 77.7 to 1.4 by 76.3 per 1000 births. This suggests that the equity gap in access to and quality of health services reduced dramatically.

### Child nutrition

**Fig. 4:**
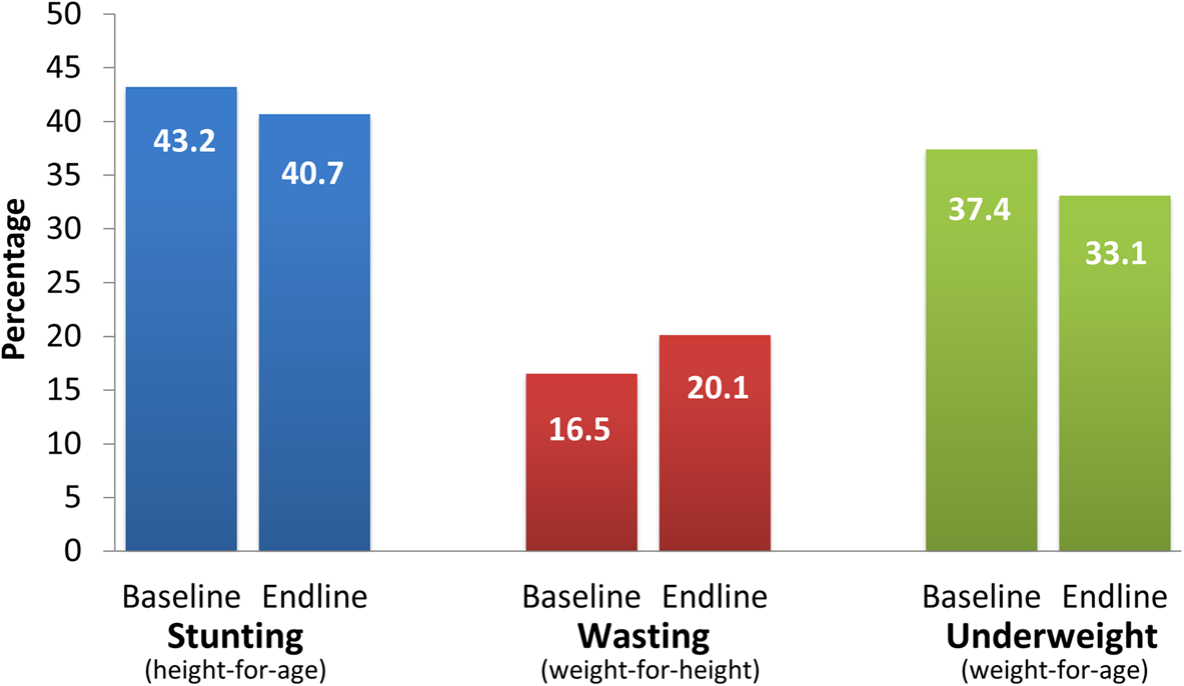
Child Nutrition in Project Areas

Analysis of child nutrition compares nutritional outcomes at baseline and endline. The prevalence of stunting (height-for-age) (Fig. 4), a measure of the cumulative effect of chronic malnutrition, reduced by 5.8% or 2.5 percentage points from 43.2% in the baseline to 40.7% in the endline. The prevalence of wasting (weight-for-height), a measure of acute or recent nutritional deficiency, increased by 21.8% or 3.6 percentage points from 16.5 to 20.1%. The prevalence of underweight (weight-for-age) children, a measure of overall nutritional health, reduced by 11.5% or 4.3 percentage points from 37.4% in the baseline to 33.1% in the endline. Prevalence of stunting and underweight decreased, although these remain high.

### Childhood diarrhea and acute respiratory infection

**Fig. 5:**
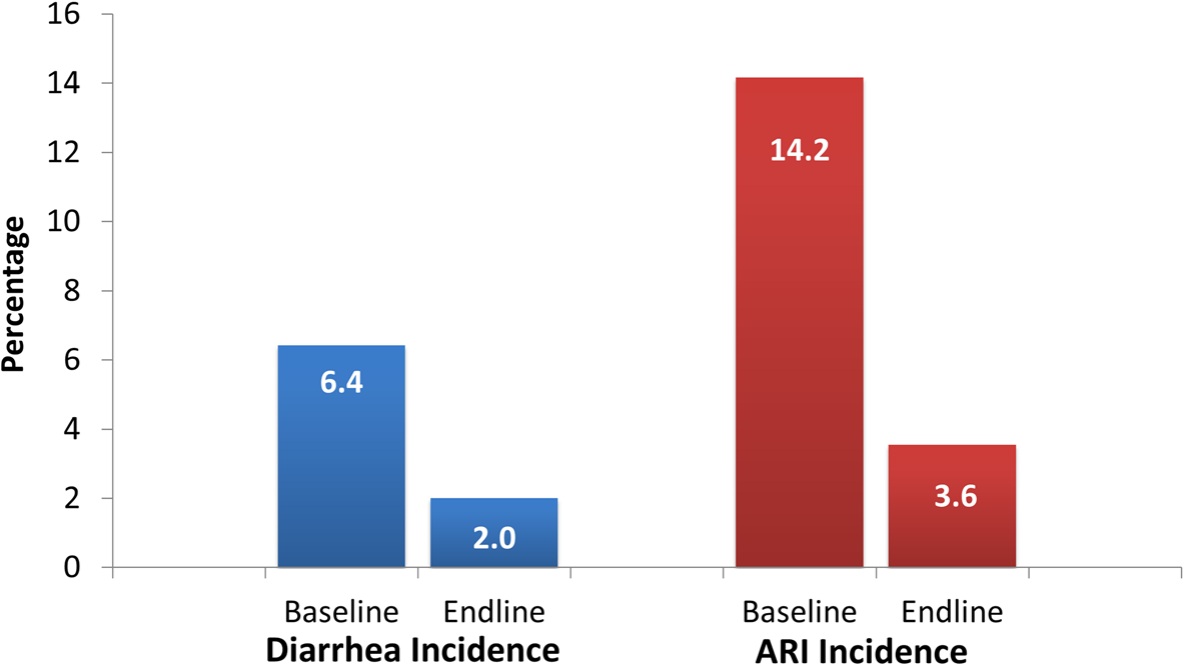
Childhood Diarrhea and ARI Prevalence in Project Areas

Childhood diarrhea prevalence was reduced from 6.4% in the baseline to 2.0% in the endline (Fig. 5) by 68.8% or 4.4 percentage points. ARI and fever prevalence experienced a large reduction from 14.2% in the baseline to 3.6% in the endline – a reduction of 74.6% or 10.6 percentage points.

The ATT of the project on the reduction of diarrhea prevalence is estimated at 3.6 percentage points and is significant (Table 2). The ATTs for ARI and fever prevalences are −2.7 and −4.1 percentage points, respectively. The combined reduction for both ARI and fever prevalence is 4.9 percentage points.

**Table 2:** Project Impact on Diarrhea, ARI and Fever Prevalence

Health Indicator	ATT		Bootstrap SE	95% CI	
				Lower	Upper
Diarrhea Prevalence	−3.6	^***^	0.9	−5.3	−1.9
ARI Prevalence	−2.7	^**^	1.3	−5.2	−0.3
Fever Prevalence	−4.1	^**^	2.0	−8.1	−0.2
ARI and Fever Prevalence	−4.9	^***^	1.1	−7.1	−2.7

Legend: ^**^ Significant at 5%; ^***^ Significant at 1%

### ANC, PNC and skilled birth attendance

**Fig. 6:**
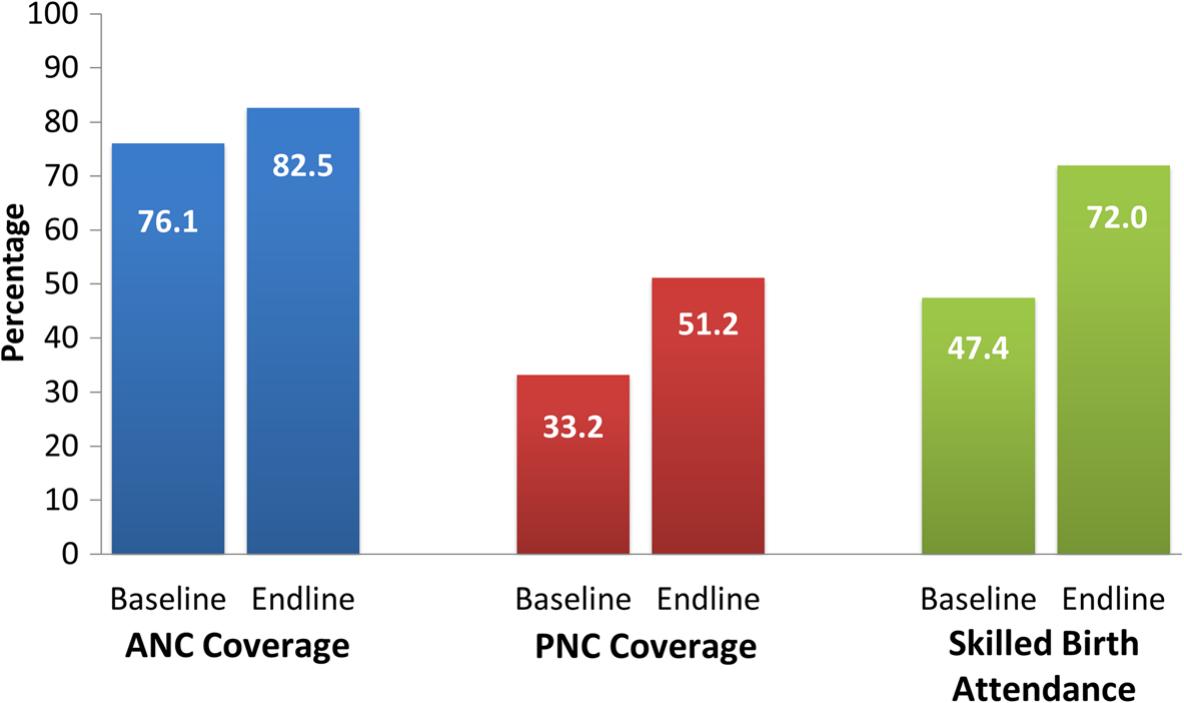
ANC, PNC and Skilled Birth Attendance in Project Areas

Pregnant women who had at least one ANC visit increased from 76.1% in the baseline to 82.5% in the endline, an 8.4% or 6.4 percentage point increase (Fig. 6). The proportion of women who received PNC increased from 33.2% in the baseline to 51.2% in the endline, a 54.2% or 18 percentage point increase. The proportion of skilled birth attendance increased from 47.4% in the baseline to 72.0% in the endline, equivalent to a 51.9% increase or 24.6 percentage points.

The number of at least three ANC visits also increased by 4.2 percentage points (Table 3). Thus, the project increased the number of ANC visits substantially from one or two visits to the recommended three visits. The project increased skilled birth attendance by 1.9 percentage points, but the effect is not significant. For PNC visits, while the trend in PA areas decreased, NPAs also had larger improvements in PNC visits than that of PAs as shown by the negative although insignificant ATT of 1.8 percentage points.

**Table 3:** Project Impact on ANC, PNC and Skilled Birth Attendance

Health Indicator	ATT		Bootstrap SE	95% CI	
				Lower	Upper
ANC Coverage (at least 1 visit)	3.3		2.1	−0.8	7.4
ANC Coverage (at least 2 visits)	2.7		2.3	−1.9	7.3
ANC Coverage (at least 3 visits)	4.2	^*^	2.5	−0.8	9.1
PNC Coverage - All mothers who gave birth within 5 years preceding the survey	−1.8		2.4	−6.6	2.9
Skilled Birth Attendance	1.9		2.4	−2.8	6.6

Notes: Last Born Child only

Legend: ^*^ Significant at 10%

### Breastfeeding

**Fig. 7:**
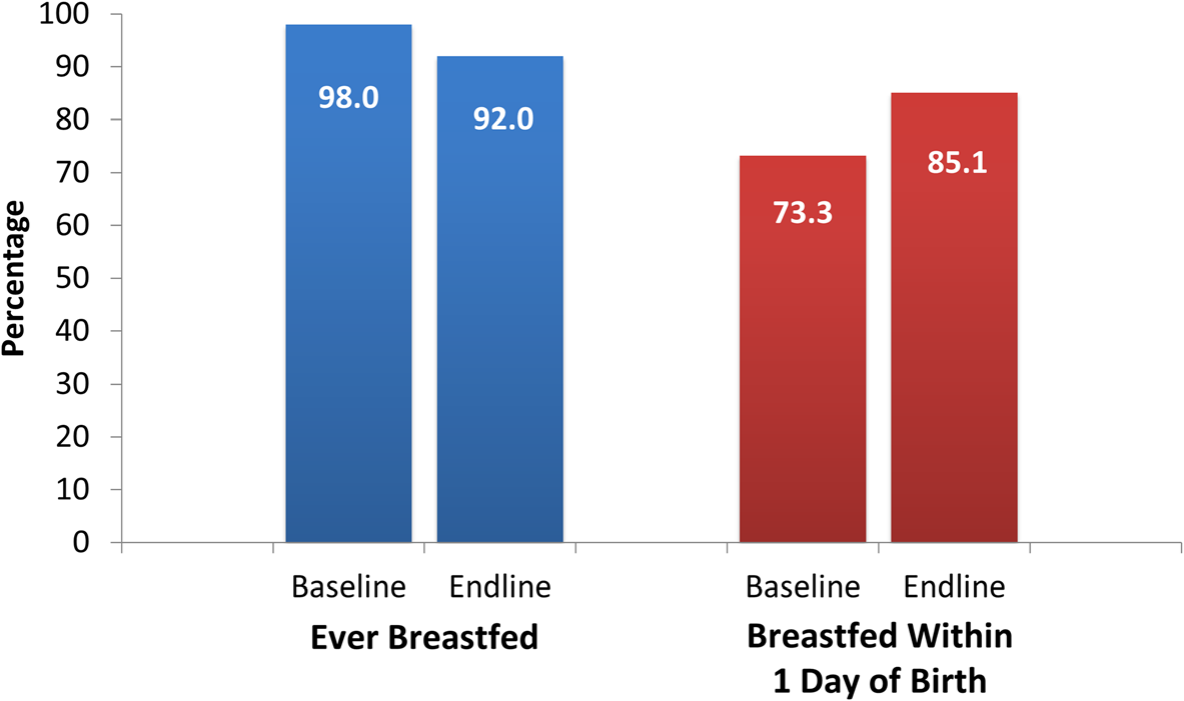
Breastfeeding Rates in Project Areas

The proportion of ever breastfed children decreased by 6.1% or 6 percentage points from 98% in the baseline to 92% in the endline (Fig. 7). The proportion of ever breastfed children was significantly reduced in PA more than in NPA, as shown by the negative and significant ATT of 3.4 percentage points. The percentage of children that were breastfed within 1 day of birth increased from 73.3% in the baseline to 85.1% in the endline but the impact is not significant (Table 4).

**Table 4:** Project Impact on Breastfeeding

Health Indicator	ATT		Bootstrap SE	95% CI	
				Lower	Upper
Ever Breastfed	−3.4	^***^	1.3	−6.0	−0.9
Breastfed within 1 day of birth	2.3		2.8	−3.1	7.7

Notes: Last Born Child only

Legend: ^***^ Significant at 1%

### Contraceptive prevalence, STI prevalence and HIV/AIDS awareness

**Fig. 8:**
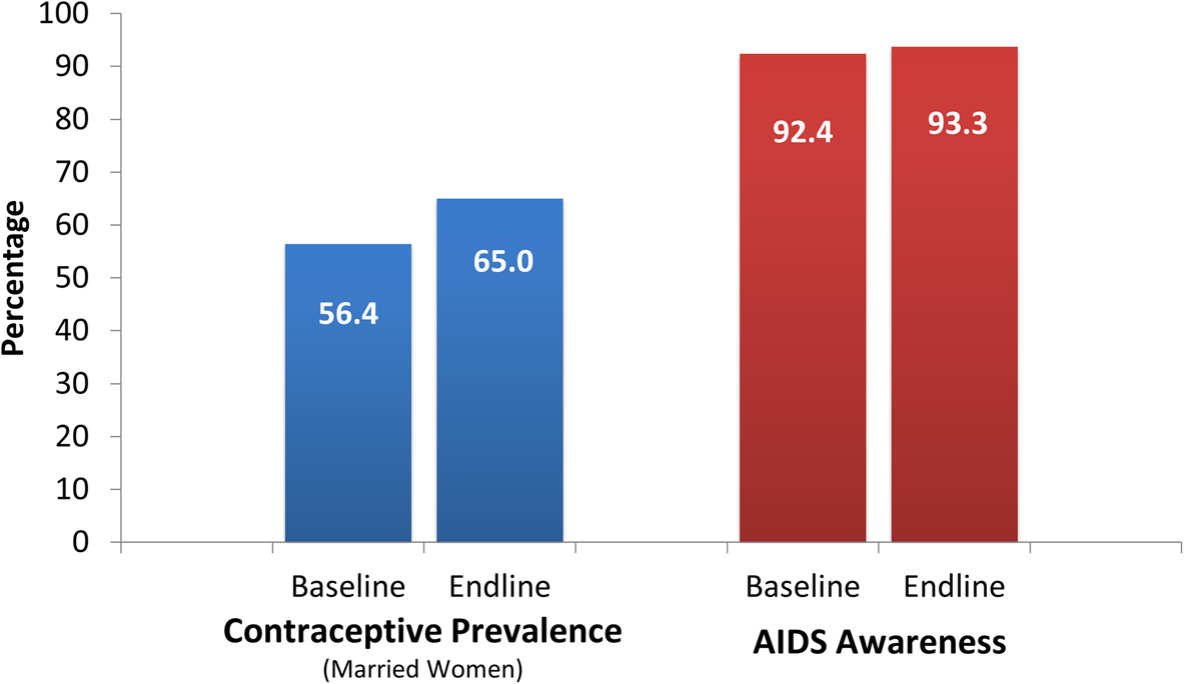
Contraceptive Prevalence Rate and HIV/AIDS Awareness in Project Areas

Use of modern contraceptive among currently married women ages 15–49 increased from 56.4 to 65% (Fig. 8). Modern methods include the use of pills, condoms, injections, IUD, female sterilization, male sterilization, and Norplant or implants. Moreover, HIV/AIDS awareness somewhat remained constant from 92.4% in the baseline to 93.3% in the endline.

The project significantly increased modern contraceptive use by around 4.5 percentage points (Table 5), and significantly reduced STI prevalence by 2.1 percentage points. The project significantly improved HIV/AIDS awareness and avoidance by 2.4 and 2.2 percentage points, respectively.

**Table 5:** Contraceptive Prevalence, STI Prevalence and HIV/AIDS Awareness

Health Indicator	ATT		Bootstrap SE	95% CI	
				Lower	Upper
Contraceptive Prevalence (married women)	4.5	^***^	1.3	2.0	7.0
STI Prevalence (women)	−2.1	^**^	1.0	−4.0	−0.2
HIV/AIDS Awareness	2.4	^***^	0.6	1.2	3.7
HIV/AIDS Avoidance	2.2	^**^	1.0	0.2	4.3

Legend: ^**^ Significant at 5%; ^***^ Significant at 1%

## Discussion

### Child-related outcomes

Several estimates of improvements in health indicators show the project’s effectiveness. Child mortality rates decreased during the project implementation period. U5MR reduction in PA was 9.5 per 1000 live births greater than that of NPA. Moreover, neonatal, infant, and child mortality rates decreased greater in PA than NPA. Despite the fact that the decrease in under-five mortality rates cannot be attributed solely to the project, the reduction of U5MR in PA compared with NPA is too large to be ignored.

There was also an improvement in the overall nutritional status of children as explained by the reduced prevalence of underweight children. Moreover, reduction in stunting and underweight prevalence is higher in PA than NPA (Additional file 1: Appendix 2). Furthermore, the increase in wasting prevalence is lower in PA than in NPA. The UHS 2013 also observed increased wasting in city corporation’s non-slum areas from 11% in 2011 to 16% in 2013, while in city corporation’s slum areas wasting prevalence remains unchanged at 19%. Feeding practices could be one of the major reasons for this increase. Increased involvement of mothers in out-of-house economic activities may hamper feeding practices of children and urban families. Wasting prevalence falls as the age of children increases. However, UHS 2013 revealed a U-shaped relationship between wasting and age of children that might be associated with feeding practices – a recent phenomenon for children under-five [20]. Still, malnutrition in Bangladesh is complex and factors range from poverty and hunger, low rates of exclusive breastfeeding, inadequate complementary feeding, and recurrent infections [21].

Despite the significant reduction of ARI from the baseline to the endline surveys, the Bangladesh Demographic Health Survey (BDHS) shows a different result in ARI incidence, which increased from 3.3 to 4.8% from 2007 to 2011. The discrepancy could be due to seasonal effects in the timing of data collection. BDHS 2007 was conducted in March to August while BDHS 2011 was conducted in July to December. UPHCP-II had a significant impact in reducing ARI and fever prevalence by around 4.9 percentage points (Table 2) through systematic case management and knowledge dissemination of ARI and fever prevention.

The reduction of 3.6 percentage points in diarrhea prevalence among children can be attributed to the wide information dissemination activities under the project. BCCM provided technical assistance to NGOs in knowledge dissemination on proper hygiene, sanitation, and proper treatment of diarrhea [17]. Treatment of diarrhea in Bangladesh by oral rehydration therapy is common and people are used to buying oral rehydration saline from pharmacies and shops. Available data show use of oral rehydration therapy is universal, although there is limited supply of oral rehydration salts from health facilities since diarrhea incidence declined substantially. UHS 2013 found that the use of open latrine in slum areas declined from 40% in 2006 to only 2% in 2013 [20].

### Mother-related outcomes

The project had a significant effect on increasing at least three ANC visits by 4.2 percentage points. The unexpected result for PNC implies that the project was less effective in following-up post-delivery care possibly due traditional norms where the mother and newborn generally stay in the home after childbirth. Outreach for PNC was also lower due to high household mobility, where around 37% of the women in the sample changed their residence when the project was being implemented. Other parallel interventions in selected NPA areas were effective, for example, evaluation of Bangladesh Rural Advancement Committee (BRAC) Manoshi found 2.4 times increase in PNC visits in BRAC project areas between 2007 and 2011 [22].

Prevalence of ever breastfed children was reduced which may be due to emerging economic opportunities in the urban areas where traditional breastfeeding practices are interrupted. All other surveys in recent years found 98% coverage of ever breastfeeding in urban areas of Bangladesh. In spite of this, breastfeeding of infant within 1 day of birth had an increasing trend.

The project also increased contraceptive prevalence rate by 4.5 percentage points, decreased STI prevalence on women by 2.1 percentage points, and increased HIV/AIDS awareness and avoidance by 2.4 and 2.1 percentage points, respectively. Pill, condom, and injectables are widely used modern methods of family planning. Subsidized social marketing and commercial sources are leading providers of these methods in urban areas. The share from UPHCP-II in providing modern methods of family planning was only about 5%. Nevertheless, the project was successful in demand creation and reproductive health message dissemination through outreach workers and counseling sessions at static facilities compared to parallel programs implemented in NPAs as there were observed improvement in HIV/AIDS awareness and avoidance. Outreach services under parallel programs in NPAs were not fully in place except in urban slum programs by BRAC.

The trend in urban TFR between baseline and endline is unexpected. Urban TFR increased from 2.26 children per woman in the baseline, to 2.61 children per woman in the endline in project areas, while it increased from 2.27 children per woman in the baseline to 2.67 children per woman in the endline. This could be due to a sizable migration flow as around 39.9 and 45.1% of the women in the PA and NPA, respectively, changed their residence from a rural to an urban area during project implementation. Rapid urbanization, mostly by migration of rural households with large household size, could have increased TFR in project areas. These migrants mostly belong to the poorest quintile and TFR among the poor is about one child more than that of the richest quintile [20]. In contrast, urban TFR, as reported in the BDHS, decreased from 2.4 in 2007 to 2.0 children per woman in 2011 [5, 23], which is lower than the replacement rate. Progress in knowledge dissemination changed the behavior of women, as seen in the increase of modern contraceptive use, knowledge of HIV/AIDS avoidance, and decreased STI prevalence, which can all be attributed to the effectiveness and impact of the project.

### Behavioral change component

The effectiveness of the project was also supported by a BCCM component which organized orientation for slum leaders, members of the ward PHC coordination committees, and local female leaders fighting violence against women; advocacy meetings for female ward commissioners and other influential women leaders; conferences for local stakeholders; and workshops for project NGOs [16]. The increase in ANC coverage and skilled birth attendance is evidence of improved follow-up by health staff and community outreach workers, and increased access and health seeking behavior due to the project’s efforts in behavior change through information, education and communication activities. Television advertisements and other promotional materials regarding the health services were also found to be effective modalities of information, but need to be improved as 15.1% of adults in the survey could recall the contents, material and messages of the campaigns [24].

The endline evaluation of the BCCM component reports that there was an increase in the visiting patterns of adults to UPHCP-II health facilities, which shows awareness of the program and heightened initiative of seeking health services [17]. There was development in pregnancy-related knowledge, prenatal and postnatal child care, vaccination, acute respiratory infection, prevention of sexually transmitted infections and improved awareness that treatment is available for such diseases. Adults who visited the health facilities had positive feedback on the quality of the service delivery, and advised their neighbors and relatives to visit the health facilities for services.

## Conclusions

The project was found to be effective in delivering health services with positive impact on various health indicators examined in terms of reduced diarrhea and acute respiratory infection in children, which could explain the downward trend in child mortality rate. Moreover, the project also improved antenatal care and skilled birth attendance. Contraceptive prevalence and HIV/AIDS awareness and avoidance increased, while sexually transmitted infection decreased.

The increase of antenatal care coverage, improvement in breastfeeding practices of women, decrease in diarrhea and acute respiratory infection prevalence, and increase in skilled birth attendance could explain the reduction of U5MR and other indicators of child mortality in project areas, and improvement in the overall nutritional health of children. Still, malnutrition in Bangladesh is complex and remains persistent with factors ranging from poverty and hunger, low rates of exclusive breastfeeding, inadequate complementary feeding, and recurrent infections [21].

Effectiveness of the project was also seen through improved quality of care as a result of a quality assurance supervisory mechanism, capacity-building activities, and health facilities established in close proximity to beneficiaries. The pro-poor delivery of health services was found to be effective due to a behavior change campaign that reduced health inequalities across wealth classes. Wide campaign coverage coupled with effective and efficient delivery of health service increased the receptivity of the beneficiary population and enhanced the impact of the project. Fees for health services for clients with capacity to pay helped NGOs meet their contractual income targets and indirectly contributed to the project’s sustainability.

Moreover, previous studies observed improved satisfaction of clients to the project. The patients expressed satisfaction with the UPHCP-II because of the close proximity of the strategically positioned health facilities to maximize accessibility. The patients also highlighted the good quality of service as another reason for their satisfaction with the project [25].

The total number of health services provided by the NGOs increased over time. It was initially observed that the cost per service was relatively high, but had decreased toward the end of the project implementation period as NGOs began to become accustomed and recognized by the community in their respective project areas [25]. Moreover, the capacity of NGOs increased as measured by the availability of staff, training, management of equipment and drugs, infection prevention, waste disposal, and use of registers [16]. These observations imply that the NGOs were becoming efficient in service delivery over time as a result of increased skill of the health personnel through capacity building and added innovations in management and implementation.

UPHCP-II was effective in improving the health status of the urban population in the project area. However, continuous efforts should be invested further to improve the nutritional status of children to reduce the prevalence of wasting. Safeguards should be set in place to actively mitigate the acute effects of malnutrition due to sudden economic shocks or political turmoil, and natural calamities. Behavior change communication and marketing activities could still be improved to increase breastfeeding practices despite the emerging economic opportunities available for women. Furthermore, PNC coverage and skilled birth attendance could still be improved, which would further reduce neonatal and infant mortality. This could be achieved by improving post-delivery health seeking behavior, quality of care, and by making sure that health services are easily accessible by the beneficiaries, particularly urban poor and mobile populations. For instance, the informal private-for-profit providers located in poor slum settlements fill an important gap in coverage where formal provision is largely absent and limited to a few NGOs providing primary care on specific days and hours of the week. Formal service delivery efforts can learn from successful strategies and prioritize the provision of affordable and quality care close to where the poor reside and at hours convenient to the working population [26].

A system of accounting all the target beneficiaries and their health status would also be vital in ensuring that the essential needs of the individual or household are provided regardless of the NGO providing the health services. This issue arises due to the mobility of project beneficiaries who transfer to or from a project area, and it could be addressed by providing each beneficiary a unique identification. The identification system could build on the existing structure of the health entitlement cards. Such a system would allow the monitoring of health status and health care utilization across different facilities in different geographic areas. It could also enable the measurement of project impact from the national or community level down to the individual or household level.

Furthermore, several primary health care projects are simultaneously present in urban Bangladesh. Increased harmonization across these projects is essential to maximize collective impact towards distributing benefits equitably across the population. Amidst rapid urbanization, new approaches to sustainable financing, efficient governance, and enhanced engagement of partners which are responsive to the dynamic conditions of the urban poor are crucial to assure greater effective coverage of comprehensive and continuous essential health services [27]. The coordination among donor institutions should include dialogue in setting clear delineation of program areas to minimize redundancy of benefits, and to fully optimize the delivery of health services. Forming an interoperable data platform across all urban health facilities from various projects would be beneficial for monitoring the health status of urban population. With this platform, improved measures of project effects can be generated as well as evidence for informing urban health policies and guiding responses to changing primary health care needs.

In order to accelerate toward universal health coverage, innovations such as government strategic purchasing of health services by contracting private and NGO sectors, high coverage of essential services, strong referral networks, strong cadre of community health workers, and ambitious experimentation and research in proven, cost-effective interventions, which were integral to Bangladesh’s impressive achievements in health need to be sustained [28]. Lessons learned from the UPHCP-II experience and this impact evaluation study are aimed at enhancing effective design, implementation, management, and delivery of health services in ongoing and future urban health interventions to achieve effective and sustainable universal health coverage.

## Additional file

Additional file 1: Supplementary tables and graphs. (DOCX 96 kb)

## Data Availability

The data that support the findings of this study are available from NIPORT but restrictions apply to the availability of these data, which were used under license for the current study, and so are not publicly available. Data are however available from the authors upon reasonable request and with permission of NIPORT.

## Abbreviations

ANC: Antenatal care
ARI: Acute respiratory infection
ATT: Average treatment effect on the treated
BCCM: Behavior change communication and marketing
CRHCC: Comprehensive reproductive health care center
DCC: Dhaka City Corporation
DHS: Demographic and health survey
NGO: Non-government organization
NIPORT: National Institute of Population Research and Training
NPA: Non-project area
OCC: other city corporations
PA: Project area
PHC: Primary health care
PNC: Postnatal care
PSM: Propensity score matching
STI: Sexually transmitted infection
TFR: Total fertility rate
U5MR: Under-5 mortality rate
UPHCP: Urban Primary Health Care Project
UPHCP-II: Second Urban Primary Health Care Project
UPHCSDP: Urban Primary Health Care Services Delivery Project

## References

[1] Bangladesh Bureau of Statistics (BBS). Bangladesh Population and Housing Census 2011 – National Report, Volume 4. Dhaka; 2012.

[2] United Nations (2014). Department of Economic and Social Affairs, population division.

[3] Afsana K, Wahid SS (2013). Health care for poor people in the urban slums of Bangladesh. Lancet.

[4] National Institute of Population Research and Training (NIPORT), MEASURE Evaluation, International Centre for Diarrhoeal Disease Research, Bangladesh, and Associates for Community and Population Research. 2006 Bangladesh Urban Health Survey. Dhaka; 2008.

[5] National Institute of Population Research and Training, Mitra and Associates, and macro international. Bangladesh demographic and health survey 2007. Dhaka; 2009.

[6] Bangladesh Bureau of Statistics. Report on the household income and expenditure survey 2010. Dhaka; 2011.

[7] Harpham T (2009). Urban health in developing countries: what do we know and where do we go?. Health Place.

[8] Asian Development Bank. Report and recommendation of the president to the Board of Directors on a proposed loan and Asian development fund Grant to the People’s republic of Bangladesh for the second urban primary health care project. Manila; 2005.

[9] Heard A, Nath DK, Loevinsohn B (2013). Contracting urban primary healthcare services in Bangladesh–effect on use, efficiency, equity and quality of care. Tropical Med Int Health.

[10] Loevinsohn B (2008). Performance-based contracting for health Services in Developing Countries.

[11] Lance P, Angeles G, Kamal N (2012). Smiling sun franchise program (BSSFP) impact evaluation report.

[12] Lagarde M, Palmer N. The impact of contracting out on health outcomes and use of health services in low and middle income countries. Cochrane Libr. 2009. 10.1002/14651858.CD008133. 10.1002/14651858.CD008133 19821443

[13] Ahmed SM, Evans TG, Standing H, Mahmud S (2013). Harnessing pluralism for better health in Bangladesh. Lancet.

[14] Arifeen S, Christou A, Reichenbach L, Osman FA, Azad K, Islam KS, Ahmed F, Perry HB, Peters DH (2013). Community-based approaches and partnerships: innovations in health-service delivery in Bangladesh. Lancet.

[15] Tanzil S, Zahidie A, Ahsan A, Kazi A, Shaikh BT (2014). A case study of outsourced primary healthcare services in Sindh, Pakistan: is this a real reform?. BMC Health Serv Res.

[16] Asian Development Bank. Completion report: Bangladesh second urban primary health care project. Manila; 2014.

[17] Bangladesh Center for Communication Programs (BCCP). End-line evaluation of the BCCM component of UPHCP-II. Dhaka; 2012.

[18] National Institute of Population Research and Training (NIPORT). End-line household survey under second urban primary health care project – Dhaka City corporation. Dhaka; 2013.

[19] Todd, P. Evaluating Social Programs with Endogenous Program Placement and Selection of the Treated, Ch. 60, pp. 3847–3894 in T. Paul Schultz and Strauss, John A. eds. Handbook of Development Economics. Amsterdam: Elsevier; 2008.

[20] National Institute of Population Research and Training (NIPORT), MEASURE Evaluation, International Centre for Diarrhoeal Disease Research, Bangladesh, and Associates for Community and Population Research. 2013 Bangladesh Urban Health Survey. Dhaka; 2015.

[21] Chowdhury AM, Bhuiya A, Chowdhury ME, Rasheed S, Hussain Z, Chen LC (2013). The Bangladesh paradox: exceptional health achievement despite economic poverty. Lancet.

[22] Alam N, Begum D, Ahmed SM, Streatfield PK. Manoshi Community Health Solutions in Bangladesh: Impact Evaluation Surveys in Dhaka Urban Slums in 2007, 2009, and 2011. Dhaka; 2011.

[23] National Institute of Population Research and Training (NIPORT), Mitra and Associates, and ICF International. Bangladesh Demographic and Health Survey 2011. Dhaka and Calverton; 2013.

[24] Bangladesh Center for Communication Programs (BCCP). Final report: second urban primary health care project. Dhaka; 2012.

[25] Asian Development Bank. Assessment of the UPHCP-II in Bangladesh. Consultant’s Report (C13461-BAN). Manila; 2012.

[26] Adams AM, Islam R, Ahmed T (2015). Who serves the urban poor? A geospatial and descriptive analysis of health services in slum settlements in Dhaka, Bangladesh. Health Policy Plan.

[27] Adams AM, Rabbani A, Ahmed S, Mahmood SS, Al-Sabir A, Rashid SF, Evans TG (2013). Explaining equity gains in child survival in Bangladesh: scale, speed, and selectivity in health and development. Lancet.

[28] Adams AM, Ahmed T, El Arifeen S, Evans TG, Huda T, Reichenbach L (2013). Innovation for universal health coverage in Bangladesh: a call to action. Lancet.

