# Supplementary material for: Impact evaluation of contracting primary health care services in urban Bangladesh
Source: BMC Health Serv Res. 2019 Nov 21;19:854. doi: 10.1186/s12913-019-4406-5 (PMC6956513; doi:10.1186/s12913-019-4406-5)
Supplement: Supplementary file 1 — Supplementary tables and graphs. (DOCX 96 kb) [file 12913_2019_4406_MOESM1_ESM.docx]

**Appendix 1**

**Sample Sizes**

| **Health Outcome** | **Indicator** | **Baseline** | | **Endline** | |
| --- | --- | --- | --- | --- | --- |
|  |  | **PA** | **NPA** | **PA** | **NPA** |
| **Infant and Child Mortality** | Neonatal Mortality | 761 | 376 | 327 | 490 |
|  | Infant Mortality (1q0) | 1,263 | 581 | 517 | 748 |
|  | Child Mortality (4q1) | 354 | 152 | 146 | 219 |
|  | U5MR (5q0) | 1,654 | 753 | 676 | 991 |
| **Total fertility rate (TFR)** | Total Fertility Rate | 7,889 | 4,034 | 9,430 | 9,598 |
| **Child Nutrition** | Stunting - cumulative effect of chronic malnutrition | 496 | 185 | 2,732 | 1,574 |
|  | Wasting - acute or recent nutritional deficit | 581 | 212 | 2,741 | 1,584 |
|  | Underweight - overall indicator of nutritional health | 593 | 217 | 2,854 | 1,657 |
| **Diarrhea and ARI** | Diarrhea Prevalence | 1,441 | 495 | 3,277 | 1,903 |
|  | ARI Prevalence | 1,441 | 495 | 3,277 | 1,903 |
|  | Fever Prevalence | 1,441 | 495 | 3,277 | 1,903 |
|  | ARI and Fever Prevalence | 1,441 | 495 | 3,277 | 1,903 |
| **Reproductive Health Care** | Skilled Birth Attendance | 2,792 | 957 | 888 | 493 |
| **Contraceptive Prevalence Rate** | Modern Contraceptive Use (married women) | 5,610 | 1,893 | 8,846 | 4,812 |
| **Sexually Transmitted Infection (STI)/ Human Immunodeficiency Virus (HIV)** | STI Prevalence (married women) | 5,610 | 1,893 | 8,846 | 4,812 |
|  | HIV/AIDS Awareness (married women) | 5,610 | 1,893 | 8,846 | 4,812 |
|  | HIV/AIDS Avoidance (married women) | 5,610 | 1,893 | 8,846 | 4,812 |
| **Breastfeeding** | Ever Breastfed | 1,077 | 387 | 888 | 493 |
|  | Breastfed within 1 day of birth | 1,077 | 387 | 888 | 493 |
| **Antenatal Care /Postnatal Care** | ANC Coverage (at least 1 visit) | 2,792 | 957 | 888 | 493 |
|  | ANC Coverage (at least 2 visits) | 2,792 | 957 | 888 | 493 |
|  | ANC Coverage (at least 3 visits) | 2,792 | 957 | 888 | 493 |
|  | PNC Coverage - All mothers who gave birth within 5 years preceding the survey | 2,792 | 957 | 888 | 493 |

Note: PA = project area, NPA = non-project area

**Appendix 2**

**Health Outcome Indicators**

| **Health Outcome** | **Indicator** | **Baseline** | | **Endline** | |
| --- | --- | --- | --- | --- | --- |
|  |  | **PA** | **NPA** | **PA** | **NPA** |
| **Infant and Child Mortality** | Neonatal Mortality | 37.9 | 33.4 | 27.9 | 28.3 |
|  | Infant Mortality (1q0) | 51.8 | 47.8 | 39.6 | 39.7 |
|  | Child Mortality (4q1) | 15.0 | 9.4 | 10.2 | 10.4 |
|  | U5MR (5q0) | 66.0 | 56.7 | 49.4 | 49.6 |
| **Total fertility rate (TFR)** | Total Fertility Rate | 2.3 | 2.3 | 2.6 | 2.7 |
| **Child Nutrition** | Stunting - cumulative effect of chronic malnutrition | 43.2 | 39.2 | 40.7 | 38.1 |
|  | Wasting - acute or recent nutritional deficit | 16.5 | 13.4 | 20.1 | 20.4 |
|  | Underweight - overall indicator of nutritional health | 37.4 | 30.6 | 33.1 | 30.7 |
| **Diarrhea and ARI** | Diarrhea Prevalence | 6.4 | 5.4 | 2.0 | 3.0 |
|  | ARI Prevalence | 14.2 | 12.5 | 3.6 | 5.1 |
|  | Fever Prevalence | 36.8 | 35.3 | 20.9 | 21.5 |
|  | ARI and Fever Prevalence | 11.7 | 10.0 | 3.4 | 4.4 |
| **Reproductive Health Care** | Skilled Birth Attendance | 47.4 | 50.0 | 72.0 | 71.3 |
| **Contraceptive Prevalence Rate** | Modern Contraceptive Use (married women) | 56.4 | 56.5 | 65.0 | 62.8 |
| **Sexually Transmitted Infection (STI)/ Human Immunodeficiency Virus (HIV)** | STI Prevalence (married women) | 31.7 | 29.7 | 3.3 | 3.6 |
|  | HIV/AIDS Awareness (married women) | 92.4 | 93.7 | 93.3 | 92.6 |
|  | HIV/AIDS Avoidance (married women) | 67.4 | 71.3 | 88.4 | 87.5 |
| **Breastfeeding** | Ever Breastfed | 98.0 | 97.9 | 92.0 | 95.2 |
|  | Breastfed within 1 day of birth | 73.3 | 75.6 | 85.1 | 88.5 |
| **Antenatal Care /Postnatal Care** | ANC Coverage (at least 1 visit) | 76.1 | 76.0 | 82.5 | 82.8 |
|  | ANC Coverage (at least 2 visits) | 66.0 | 65.7 | 74.2 | 73.8 |
|  | ANC Coverage (at least 3 visits) | 51.7 | 52.7 | 59.9 | 56.2 |
|  | PNC Coverage - All mothers who gave birth within 5 years preceding the survey | 33.2 | 30.8 | 51.2 | 48.4 |

Note: PA = project area, NPA = non-project area

**Appendix 3**

**Table A3.1. Logit Model Results - Child Baseline**

| Variables | Coef. | | Robust SE | 95% CI | |
| --- | --- | --- | --- | --- | --- |
|  |  |  |  | Lower | Upper |
| Wealth Index | -0.031 | * | 0.017 | -0.063 | 0.002 |
| Age in Months | -0.001 |  | 0.002 | -0.005 | 0.003 |
| Gender (Male=1) | -0.118 | * | 0.065 | -0.244 | 0.009 |
| Parity | -0.016 |  | 0.022 | -0.058 | 0.027 |
| Education of Mother (Primary) | -0.180 | ** | 0.086 | -0.349 | -0.012 |
| Education of Mother (Secondary) | -0.199 | ** | 0.099 | -0.394 | -0.004 |
| Education of Mother (College+) | -0.129 |  | 0.150 | -0.424 | 0.166 |
| Religion (Hinduism) | 0.473 | *** | 0.151 | 0.178 | 0.769 |
| Religion (Others) | -0.041 |  | 0.427 | -0.878 | 0.796 |
| Area (Other City Corporations) | 1.093 | *** | 0.067 | 0.962 | 1.225 |
| Area (Municipalities) | -0.841 | *** | 0.228 | -1.287 | -0.395 |
| Constant | 0.239 | ** | 0.120 | 0.004 | 0.474 |
| N=4,699; Wald ChiSq=352.79(p-value<0.01); Pseudo RSq=0.0621; Log-Likelihood=-3,027.36 | | | | | |
| Base Categories: No Education; Islam Religion; Dhaka City Corporation | | | | | |

**Table A3.2. Logit Model Results - Woman Baseline**

| Variables | Coef. | | Robust SE | 95% CI | |
| --- | --- | --- | --- | --- | --- |
|  |  |  |  | Lower | Upper |
| Wealth Index | -0.003 |  | 0.010 | -0.023 | 0.017 |
| Age in Months | -0.002 |  | 0.002 | -0.006 | 0.003 |
| Parity | 0.013 |  | 0.012 | -0.010 | 0.036 |
| Education of Mother (Primary) | -0.005 |  | 0.056 | -0.116 | 0.105 |
| Education of Mother (Secondary) | -0.137 | ** | 0.062 | -0.258 | -0.016 |
| Education of Mother (College+) | -0.243 | *** | 0.084 | -0.407 | -0.079 |
| Religion (Hinduism) | 0.375 | *** | 0.081 | 0.216 | 0.533 |
| Religion (Others) | 0.056 |  | 0.224 | -0.383 | 0.494 |
| Area (Other City Corporations) | 0.885 | *** | 0.042 | 0.802 | 0.968 |
| Area (Municipalities) | -0.777 | *** | 0.133 | -1.036 | -0.517 |
| Constant | 0.220 | ** | 0.087 | 0.049 | 0.392 |
| N=11,585; Wald ChiSq=607.25(p-value<0.01); Pseudo RSq=0.0424; Log-Likelihood=-7,090.12 | | | | | |
| Base Categories: No Education; Islam Religion; Dhaka City Corporation | | | | | |

**Table A3.3. Logit Model Results - Child Endline**

| Variables | Coef. | | Robust SE | 95% CI | |
| --- | --- | --- | --- | --- | --- |
|  |  |  |  | Lower | Upper |
| Wealth Index | 0.003 |  | 0.013 | 0.220 | 0.823 |
| Age in Months | -0.002 |  | 0.001 | -1.500 | 0.134 |
| Gender (Male=1) | 0.068 |  | 0.047 | 1.450 | 0.146 |
| Parity | -0.099 | *** | 0.021 | -4.790 | 0.000 |
| Education of Mother (Primary) | 0.080 |  | 0.087 | 0.920 | 0.356 |
| Education of Mother (Secondary) | -0.165 | * | 0.087 | -1.900 | 0.058 |
| Education of Mother (College+) | -0.090 |  | 0.106 | -0.860 | 0.392 |
| Religion (Hinduism) | 0.543 | *** | 0.089 | 6.110 | 0.000 |
| Religion (Others) | -0.103 |  | 0.416 | -0.250 | 0.805 |
| Area (Other City Corporations) | 0.046 |  | 0.057 | 0.810 | 0.419 |
| Area (Municipalities) | -0.175 | *** | 0.062 | -2.810 | 0.005 |
| Constant | 0.224 | ** | 0.109 | 2.050 | 0.040 |
| N=7,385; Wald ChiSq=97.62(p-value<0.01); Pseudo RSq=0.0098; Log-Likelihood=-5,065.03 | | | | | |
| Base Categories: No Education; Islam Religion; Dhaka City Corporation | | | | | |

**Table A3.4. Logit Model Results - Woman Endline**

| Variables | Coef. | | Robust SE | 95% CI | |
| --- | --- | --- | --- | --- | --- |
|  |  |  |  | Lower | Upper |
| Wealth Index | 0.018 | ** | 0.008 | 0.002 | 0.034 |
| Age in Months | 0.004 | * | 0.002 | 0.000 | 0.009 |
| Parity | -0.092 | *** | 0.013 | -0.118 | -0.066 |
| Education of Mother (Primary) | 0.039 |  | 0.049 | -0.057 | 0.136 |
| Education of Mother (Secondary) | -0.054 |  | 0.049 | -0.149 | 0.042 |
| Education of Mother (College+) | -0.056 |  | 0.060 | -0.174 | 0.061 |
| Religion (Hinduism) | 0.498 | *** | 0.051 | 0.399 | 0.598 |
| Religion (Others) | 0.344 |  | 0.243 | -0.132 | 0.820 |
| Area (Other City Corporations) | -0.010 |  | 0.035 | -0.079 | 0.059 |
| Area (Municipalities) | -0.046 |  | 0.039 | -0.122 | 0.029 |
| Constant | 0.031 |  | 0.081 | -0.128 | 0.190 |
| N=19,042; Wald ChiSq=177.86(p-value<0.01); Pseudo RSq=0.007; Log-Likelihood=-13,106.5 | | | | | |
| Base Categories: No Education; Islam Religion; Dhaka City Corporation | | | | | |

**Appendix 4**

**Table A4.1. Woman Propensity Scores**

| Descriptive Statistics | Baseline | | | Endline | | |
| --- | --- | --- | --- | --- | --- | --- |
|  | PA | NPA | Total | PA | NPA | Total |
| Mean | 0.6814 | 0.6266 | 0.6629 | 0.5005 | 0.4909 | 0.4956 |
| SD | 0.1016 | 0.1192 | 0.1109 | 0.0500 | 0.0473 | 0.0489 |
| Skewness | -1.1159 | -0.4767 | -0.8814 | 0.9874 | 0.8241 | 0.9155 |
| Kurtosis | 3.2449 | 2.3571 | 2.8041 | 4.4092 | 5.5077 | 4.9415 |
| Min | 0.2979 | 0.2925 | 0.2925 | 0.3083 | 0.2869 | 0.2869 |
| Max | 0.8212 | 0.8209 | 0.8212 | 0.6833 | 0.6868 | 0.6868 |

**Table A4.2. Child Propensity Scores**

| Descriptive Statistics | Baseline | | | Endline | | |
| --- | --- | --- | --- | --- | --- | --- |
|  | PA | NPA | Total | PA | NPA | Total |
| Mean | 0.6596 | 0.5878 | 0.6361 | 0.4066 | 0.3907 | 0.3983 |
| SD | 0.1267 | 0.1474 | 0.1380 | 0.0841 | 0.0835 | 0.0842 |
| Skewness | -0.9893 | -0.3185 | -0.7551 | 0.1842 | 0.0998 | 0.1409 |
| Kurtosis | 2.7604 | 2.0376 | 2.3929 | 2.9095 | 2.9419 | 2.9438 |
| Min | 0.1841 | 0.1844 | 0.1841 | 0.1603 | 0.1411 | 0.1411 |
| Max | 0.8657 | 0.8624 | 0.8657 | 0.7020 | 0.6992 | 0.7020 |

**Appendix 5**

**Balancing Test and Regions of Common Support for Nearest Neighbor Matching**

| **Table A5.1. Balancing Test - Children PSM at Baseline** | | | | | |
| --- | --- | --- | --- | --- | --- |
| Variable | Mean | | %Bias | t-test | |
|  | Treated | Control |  | t | p>t |
| Wealth Index | -0.1414 | -0.0616 | -3.20 | -0.840 | 0.399 |
| Age in Months | 28.4980 | 29.5500 | -6.00 | -1.610 | 0.108 |
| Gender (Male=1) | 0.4983 | 0.4831 | 3.00 | 0.810 | 0.417 |
| Parity | 1.5017 | 1.5507 | -6.30 | -1.720 | 0.086 |
| Education of Mother (Primary) | 0.2838 | 0.2738 | 2.20 | 0.600 | 0.550 |
| Education of Mother (Secondary) | 0.3456 | 0.3287 | 3.50 | 0.960 | 0.336 |
| Education of Mother (College+) | 0.1423 | 0.1656 | -6.60 | -1.730 | 0.083 |
| Religion (Hinduism) | 0.0951 | 0.0941 | 0.40 | 0.090 | 0.929 |
| Religion (Others) | 0.0063 | 0.0047 | 2.00 | 0.550 | 0.579 |
| Area (Other City Corporations) | 0.7370 | 0.7370 | 0.00 | 0.000 | 1.000 |
| Area (Municipalities) | 0.0139 | 0.0139 | 0.00 | 0.000 | 1.000 |

Base Categories: No Education; Islam Religion; Dhaka City Corporation

| **Table A5.2. Balancing Test - Woman PSM Baseline** | | | | | |
| --- | --- | --- | --- | --- | --- |
| Variable | Mean | | %Bias | t-test | |
|  | Treated | Control |  | t | p>t |
| Wealth Index | -0.1739 | -0.1545 | -0.80 | -0.290 | 0.771 |
| Age | 26.4470 | 26.7830 | -5.60 | -2.090 | 0.037 |
| Parity | 2.4516 | 2.5110 | -3.70 | -1.370 | 0.171 |
| Education (Primary) | 0.2701 | 0.3093 | -8.80 | -3.240 | 0.001 |
| Education (Secondary) | 0.2951 | 0.2950 | 0.00 | 0.010 | 0.991 |
| Education (College+) | 0.1121 | 0.1109 | 0.40 | 0.140 | 0.885 |
| Religion (Hinduism) | 0.0856 | 0.0846 | 0.40 | 0.130 | 0.893 |
| Religion (Others) | 0.0065 | 0.0051 | 1.70 | 0.670 | 0.502 |
| Area (Other City Corporations ) | 0.7375 | 0.7375 | 0.00 | 0.000 | 1.000 |
| Area (Municipalities) | 0.0093 | 0.0093 | 0.00 | 0.000 | 1.000 |

Base Categories: No Education; Islam Religion; Dhaka City Corporation

| **Table A5.3. Balancing Test - Children PSM at Endline** | | | | | |
| --- | --- | --- | --- | --- | --- |
| Variable | Mean | | %Bias | t-test | |
|  | Treated | Control |  | t | p>t |
| Wealth Index | -0.2019 | -0.1775 | -1.00 | -0.410 | 0.682 |
| Age in Months | 29.3770 | 29.3500 | 0.20 | 0.060 | 0.950 |
| Gender (Male=1) | 0.5034 | 0.5141 | -2.10 | -0.840 | 0.400 |
| Parity | 2.0161 | 1.9600 | 4.60 | 1.950 | 0.051 |
| Education of Mother (Primary) | 0.2255 | 0.2147 | 2.70 | 1.030 | 0.304 |
| Education of Mother (Secondary) | 0.4429 | 0.4500 | -1.40 | -0.560 | 0.575 |
| Education of Mother (College+) | 0.2184 | 0.2251 | -1.60 | -0.630 | 0.526 |
| Religion (Hinduism) | 0.1010 | 0.0950 | 2.20 | 0.800 | 0.423 |
| Religion (Others) | 0.0035 | 0.0027 | 1.50 | 0.590 | 0.554 |
| Area (Other City Corporations) | 0.4027 | 0.3988 | 0.80 | 0.310 | 0.756 |
| Area (Municipalities) | 0.2609 | 0.2647 | -0.90 | -0.350 | 0.730 |

Base Categories: No Education; Islam Religion; Dhaka City Corporation

| **Table A5.4 Balancing Test - Woman PSM at Endline** | | | | | |
| --- | --- | --- | --- | --- | --- |
| Variable | Mean | | %Bias | t-test | |
|  | Treated | Control |  | t | p>t |
| Wealth Index | -0.1710 | -0.1042 | -2.80 | -0.610 | 0.544 |
| Age | 25.2590 | 24.8780 | 6.90 | 1.500 | 0.135 |
| Parity | 1.7601 | 1.7250 | 3.20 | 0.740 | 0.460 |
| Education (Primary) | 0.2016 | 0.1926 | 2.30 | 0.480 | 0.633 |
| Education (Secondary) | 0.4673 | 0.4984 | -6.20 | -1.310 | 0.190 |
| Education (College +) | 0.2365 | 0.2248 | 2.80 | 0.590 | 0.558 |
| Religion (Hinduism) | 0.1160 | 0.1117 | 1.50 | 0.280 | 0.777 |
| Religion (Others) | 0.0045 | 0.0077 | -5.10 | -0.850 | 0.393 |
| Area (Other City Corporations) | 0.4110 | 0.3741 | 7.60 | 1.590 | 0.111 |
| Area (Municipalities) | 0.2534 | 0.2649 | -2.60 | -0.550 | 0.581 |

Base Categories: No Education; Islam Religion; Dhaka City Corporation

**Figure A5.1. Baseline Propensity Score Distributions**

| **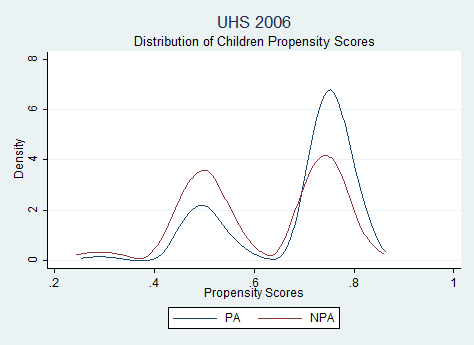** | 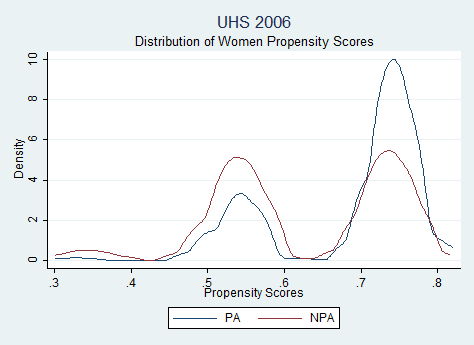 |
| --- | --- |

**Figure A5.2. Endline Propensity Score Distributions**

| **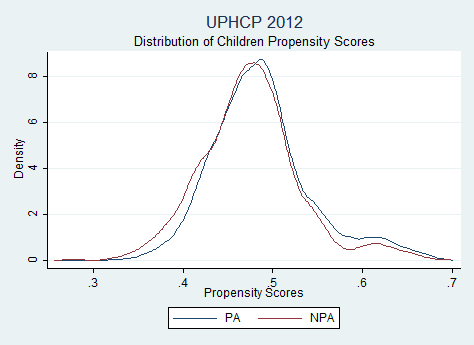** | **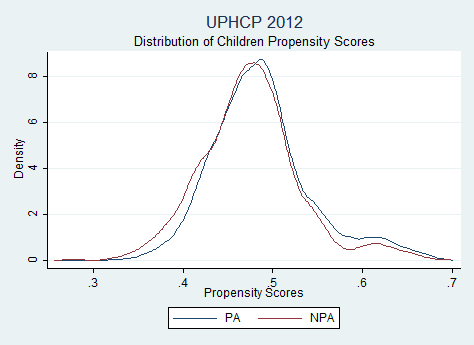** |
| --- | --- |
